# Cross-sectional assessment of body composition and detection of malnutrition risk in participants with low body mass index and eating disorders using 3D optical surface scans

**DOI:** 10.1016/j.ajcnut.2023.08.004

**Published:** 2023-08-19

**Authors:** Andrea K. Garber, Jonathan P. Bennett, Michael C. Wong, Isaac Y. Tian, Gertraud Maskarinec, Samantha F. Kennedy, Cassidy McCarthy, Nisa N. Kelly, Yong E. Liu, Vanessa I. Machen, Steven B. Heymsfield, John A. Shepherd

**Affiliations:** 1 Department of Pediatrics, University of California, San Francisco, CA, United States; 2 Graduate Program in Human Nutrition, University of Hawai’i Manoa, Honolulu, HI, United States; 3 University of Hawai’i Cancer Center, Honolulu, HI, United States; 4 Paul G. Allen School of Computer Science and Engineering, University of Washington, Seattle, WA, United States; 5 Pennington Biomedical Research Center, Louisiana State University, Baton Rouge, LA, United States

**Keywords:** 3-Dimensional Optical imaging (3DO), anorexia nervosa (AN), atypical anorexia nervosa (atypical AN), adolescents, body composition, malnutrition, sarcopenia, nutritional rehabilitation, body mass index (BMI), dual X-ray absorptiometry (DXA)

## Abstract

**Background:**

New recommendations for the assessment of malnutrition and sarcopenia include body composition, specifically reduced muscle mass. Three-dimensional optical imaging (3DO) is a validated, accessible, and affordable alternative to dual X-ray absorptiometry (DXA).

**Objective:**

Identify strengths and weaknesses of 3DO for identification of malnutrition in participants with low body mass index (BMI) and eating disorders.

**Design:**

Participants were enrolled in the cross-sectional Shape Up! Adults and Kids studies of body shape, metabolic risk, and functional assessment and had BMI of <20 kg/m^2^ in adults or <85% of median BMI (mBMI) in children and adolescents. A subset was referred for eating disorders evaluation. Anthropometrics, scans, strength testing, and questionnaires were completed in clinical research centers. Lin’s Concordance Correlation Coefficient (CCC) assessed agreement between 3DO and DXA; multivariate linear regression analysis examined associations between weight history and body composition.

**Results:**

Among 95 participants, mean ± SD BMI was 18.3 ± 1.4 kg/m^2^ in adult women (*N* = 56), 19.0 ± 0.6 in men (*N* = 14), and 84.2% ± 4.1% mBMI in children (*N* = 25). Concordance was excellent for fat-free mass (FFM, CCC = 0.97) and strong for appendicular lean mass (ALM, CCC = 0.86) and fat mass (FM, CCC = 0.87). By DXA, 80% of adults met the low FFM index criterion for malnutrition, and 44% met low ALM for sarcopenia; 52% of children and adolescents were <−2 z-score for FM. 3DO identified 95% of these cases. In the subset, greater weight loss predicted lower FFM, FM, and ALM by both methods; a greater percentage of weight regained predicted a higher percentage of body fat.

**Conclusions:**

3DO can accurately estimate body composition in participants with low BMI and identify criteria for malnutrition and sarcopenia. In a subset, 3DO detected changes in body composition expected with weight loss and regain secondary to eating disorders. These findings support the utility of 3DO for body composition assessment in patients with low BMI, including those with eating disorders.

This trial was registered at clinicaltrials.gov as NCT03637855.

## Introduction

Malnutrition increases the risk for medical complications and worsens the prognosis for a range of illnesses [[1], [2], [3], [4]], burdening healthcare systems [5] and inflating health care costs [5,6]. Malnutrition is associated with impaired physical function (e.g., low strength) and morbidity in aging populations [7] that increase the risk of hospitalization more than 4-fold [5]. Once hospitalized, malnutrition increases mortality risk 7-fold [1]. In response, new definitions of malnutrition, including assessment of body composition, were recently established in adults [[8], [9], [10]], and consensus is building in children and adolescents [11,12]. Low-muscle mass is now recognized as a key phenotypic indicator of malnutrition and sarcopenia and has been shown to greatly increase the power of nutrition screening to detect risk for mortality [[2], [3], [4]] and medical complications [1,13]. If an etiologic criterion is met (e.g., reduced food intake), malnutrition can be diagnosed by low fat-free mass index (FFMI), low appendicular lean mass (ALM), or low ALM index (ALMI) [8,10]. If strength is impaired (e.g., low handgrip), sarcopenia can be diagnosed by low ALM or ALMI [14].

Youth with anorexia nervosa (AN) comprise a unique population with malnutrition and well characterized changes in body composition. Despite advances in treatment, AN remains the deadliest mental illness, with mortality rates similar to childhood cancers [15] and among the top 6 most costly pediatric primary mental health diagnoses [16]. Studies using dual-energy X-ray absorptiometry (DXA) in patients with AN show reduced overall fat-free mass (FFM) and diminished fat mass (FM), with selective loss from the extremities. This pattern of malnutrition is associated with short-term medical complications requiring hospitalization [[17], [18], [19]]. Long-term complications related to incomplete recovery of FFM in AN [20] include persistent deficiencies in strength and physical functioning [[21], [22], [23]]. FM is not included in the new definitions of malnutrition, which are aimed at hospitalized patients and, more broadly, at chronic disease. However, in AN, reduced body fat is associated with short-term medical complications [24,25] and poor long-term outcomes, including relapse [26,27] and persistent amenorrhea [26].

Although body composition provides great insight into the assessment of malnutrition that weight alone cannot, several barriers prevent the routine assessment of body composition in clinical care. DXA is the most widely used criterion method and is considered safe [28,29]. However, governing bodies, such as the Medical and Scientific Commission of the International Olympic Committee and the International Society for Clinical Densitometry caution against >2 scans per year in children [28] and 4 per year in adults [29] due to cumulative radiation dose. Further diminishing the feasibility in most clinical settings, DXA instrumentation is large and costly. On the other hand, 3-dimensional body shape has been shown to accurately predict total and regional body composition [30–34)]. With the advent of 3-dimensional whole-body optical (3DO) surface scanning, accurate, precise, and inexpensive measures of body composition can be obtained without ionizing radiation. We have demonstrated the accuracy and precision of 3DO for the assessment of fat and lean composition in children, adolescents [30,31], and adults [32] across ethnicities [33] in relation to strength and biomarkers [34] and for monitoring changes in body composition during the course of intervention [35]. The purpose of the present study was to identify the strengths and weaknesses of 3DO for the assessment of body composition in participants with low BMI (kg/m^2^) compared with DXA. Secondarily, we sought to examine whether 3DO could predict expected changes in body composition based on weight history (loss and gain) in a subset of adolescents and young adults referred for evaluation of malnutrition secondary to eating disorders.

## Methods

### Participants

Participants were part of the larger Shape Up! cross-sectional studies designed to explore the associations of body shape to common metabolic risk factors, including body composition functional assessment and blood biomarkers: Shape Up! Kids (NIH R01DK111698, clinicaltrials.gov ID NCT03706612) and Shape Up! Adults (NIH R01DK109008, clinicaltrials.gov ID NCT03637855). Shape Up! enrolled a diverse sample of 883 adults and 465 healthy children and adolescents (aged 5–17) stratified by sex, age, ethnicity, BMI (Adults) or BMI z-score (Kids), and study site (see Supplementary Figure 1). A total of 70 adults and 25 children/adolescents who enrolled in Shape Up! were included in the present study (total *N* = 95) if BMI was low, defined as <20 kg/m^2^ in adults (>18 y old) or <85% of the median BMI (mBMI) for age and sex in children and adolescents [36]. Among these 95 participants, 15 who were referred for evaluation of malnutrition to our Eating Disorders Program and enrolled in Shape Up! studies were included at any BMI because they met the phenotypic criterion of weight loss for adults [8,9,37] or children [38]. Participants self-reported their ethnicity from the 5 ethnic subgroups (non-Hispanic White [White], non-Hispanic Black [Black], Hispanic, Asian, and Native Hawaiian or Other Pacific Islander. Weight loss was assessed by a self-report questionnaire and confirmed by a growth curve (see method below).

Data were collected in 3 clinical research centers: San Francisco, CA; Baton Rouge, LA; and Honolulu, HI. Methods have been previously described in detail [[30], [32], [34]]. Participants were recruited from October 2016 to January 2020 and provided informed consent (adults) or assent with parental consent (children and adolescents). The study protocols were approved by the institutional review board (IRB) at the 3 study sites, University of California, San Francisco (UCSF IRB #16-20197 [kids]; #15-18066 [adults]); the University of Hawaii Cancer Center (UHCC), (UHCC IRB #24282 [kids]; #01018 [adults]); and Pennington Biomedical Research Center (PBRC IRB #2017- 10, Federalwide Assurance #00006218 [kids]; #2016-053 [adults]).

### Anthropometry

Height and weight were measured in triplicate, digitally with wireless transmission (Seca 284, Seca N. America); the average reading was recorded. A special protocol was developed to avoid psychologic triggering of participants with eating disorders that included blinded (backward) weighing and obfuscated scan images. BMI was calculated from measured heights and weights. For participants aged <18 y, %mBMI was calculated as BMI divided by the 50th percentile BMI for age and sex [36]; BMI of 22 kg/m^2^ was used as the median for adults.

### Weight history

In the subset, weight history prior to referral to the Eating Disorder Program was assessed by a proctored survey and corroborated with historical height, weight, and growth charts in the medical record. These data were used to calculate the amount, duration, and rate of weight loss and weight regain. The amount of weight loss was calculated as magnitude (kg) and percent of body mass ([highest weight – lowest weight]/highest weight). The rate of weight loss was calculated as kg of weight lost over duration of loss (defined as the number of months between the highest and lowest recorded weights). The period between referral to the Eating Disorder Program, enrollment in the Shape Up! trial, and the study visit varied among participants. We calculated the amount of weight regained during this period in kg (weight at study visit – lowest weight) and percent (kg regained/kg lost).

### 3DO Scans

3DO scans were taken in duplicate using a whole-body scanner (Fit3D ProScanner version 4.X, Fit3D Inc.). Standardized body positioning was used [32]. Briefly, participants wearing form-fitting garments and swim caps stood while grasping handles on a rotating platform with a weight measurement plate and 3 infrared cameras built into a tower. Full body scan took ∼45 s during which light-coding depth sensors captured the 3D shape as the participant rotated. The resulting outputs included a 3D mesh and automated anthropometry, which included lengths, circumferences, volumes, and surface areas. Body composition for children and adolescents was calculated using equations derived from the automated anthropometry described in Wong et al. 2019 [30]; body composition for adults was derived from the 3D mesh using methods described in Wong et al. 2021 [32]. In summary, the raw 3D meshes were registered to a 110,000-vertex template mesh and reposed to a T-pose (arms horizontal to torso and posture up-right). The registered meshes were transformed by a statistical shape model into principal components, which were used to derive body composition outcomes.

### DXA scans

Whole-body DXA scan was performed once on a Hologic Discovery/A system (Hologic Inc.). Per manufacturer protocol, participants were centered on the scan table with arms at their sides, hands pronated, and feet in a plantar flexed position [39]. Full scans took ∼3 min and provided total body mass, total and regional (trunk, arms, and legs) FM, bone mineral content, and FFM. DXA cross-calibration phantoms were circulated between all sites, and calibration equations were derived to remove systematic bias in all bone and soft tissue results. DXA scans were analyzed centrally at the University of Hawaii Cancer Center by a trained technologist using Hologic Apex version 5.6 with the NHANES Body Composition Analysis calibration option disabled. Body composition was estimated as follows: fat mass index (FMI) and FFMI were calculated by dividing FM and FFM by height squared. Percent body fat (PBF) by 3DO was FM/scale weight. ALM by both methods was calculated as (arm + leg FFM) ∗ 2. ALMI was calculated as ALM/(height squared) or ALM/BMI where indicated.

### Strength testing

Strength was measured with a handgrip dynamometer for the left and right hand (JAMAR 5030J1; Sammons Preston Rolyan). With elbow at 90º flexion, participants squeezed the dynamometer as hard as they could and were then encouraged to squeeze even harder. Strength was measured in kilograms; the average of 3 measurements was taken, and the highest value obtained by either hand was used for analysis.

### Statistical methods

Summary statistics described participants by sex and age. Lin’s Concordance Correlation Coefficient (CCC) [40] assessed the agreement between 3DO and DXA (the criterion measure) to improve the clarity of comparisons. Mean differences between methods were assessed with a paired t-test, alpha 0.05 for significance. Interrater reliability between DXA and 3DO was determined by Cohen’s Kappa, which compares the observed agreement for the 2 methods (the proportion of cases for which the DXA and 3DO agree) to the expected agreement (the proportion of agreements that would be expected by chance) [41]. Linear regression with coefficient of determination and Bland-Altman plots illustrated the differences between 3DO and DXA. Studentized residual and leverage plots were used to identify outliers (not shown). Of the total eligible 99 participants, 4 scan results were excluded based on our a priori exclusion criterion of >2 SD difference between predicted (3DO) and reference measure (DXA), leaving a final sample of *N* = 95. This sample size provided adequate power to detect differences between the 2 methods: estimating sample size by 2-sample paired-means test using a standard significance level of 0.05 and power of 80%, a sample of *N* = 73 would have been adequate to detect the difference between DXA and 3DO found for the main outcome of FFM (-0.6 ± 1.8, see results), and a sample of *N* = 93 would have provided sufficient power to detect the smallest difference between methods (0.5 ± 1.7 for FM). Pediatric z-scores for FMI and FFMI were calculated using an online age- and height-based calculator [42]. Multivariable linear regression models examined the relationship between body composition by 3DO (FFMI and ALMI as predictors in separate equations) and handgrip strength (outcome), adjusted for BMI and age. In the subset (*N* = 15), comparisons with the larger sample of participants with low BMI were made by unpaired t-tests. Relationships between weight history (percent of body mass lost or regained as predictors) and body composition (outcomes) were examined with multivariable linear regression models in both unadjusted models and models adjusted for the highest historical %mBMI. Analyses were performed using STATA version 16.1 (StataCorp).

## Results

### Participants characteristics

Table 1 shows participant characteristics, selected markers of nutritional status and body composition by DXA, and criteria for diagnosis of malnutrition and sarcopenia. Among 95 ethnically diverse participants with low BMI, *N* = 70 were adults: 80% were women aged 18–89 y, and 20% were men aged 19–74 y. Virtually all adults (97% [68/70]) had low BMI, defined as <20 kg/m^2^ [8]. Lean mass was low, with an average FFMI below the threshold for diagnosis of malnutrition [8]. (See below for the number of participants meeting the criteria for malnutrition and sarcopenia.) PBF was 24.3% ± 4.8% in female adults and 15.4 ± 4.1 in male. Also shown in Table 1 are the 25 children and adolescents aged 7–17 y. Malnutrition was mild on average based on BMI (39); average z-scores (SD) for FMI and FFMI were in the range of −1 to −2 z-score.

**TABLE 1:** Participant characteristics, selected markers of nutritional status and body composition by DXA, percent meeting criteria for malnutrition and sarcopenia

Characteristics at study visit (all, *N* = 95)	Malnutrition or sarcopenia criterion	Mean ± SD or % (No.)	Percent meeting criterion by DXA(%)
Age		36.1 ± 21.2	
Female		74% (70)	
Ethnicity			
Non-Hispanic White		45.3% (43)	
Asian		27.4% (26)	
Non-Hispanic Black		10.5% (10)	
Hispanic		11.6% (11)	
NHOPI		5.3% (5)	
**Female adults (≥18 y, *N* = 56)**			
Age (y)		45.4 ± 18.9	
BMI (kg/m^2^)	<20^1^	18.3 ± 1.4	96% (54)
FFMI (kg/m^2^)	<15^1^^,^^2^	13.8 ± 1.1	82% (46)
ALM (kg)	<14.1^1^ or < 15^3^	15.4 ± 2.3	48% (27)
ALMI (kg/m^2^)	<5.25^1^ or <5.5^3^	5.7 ± 0.6	41% (23)
ALM adjusted for BMI (ALM/BMI)	<0.591^1^	0.84 ± 0.11	2% (1)
Grip strength (kg)	<16 3	19.4 ± 7.2	30% (17)
PBF (%)	N/A	24.3 ± 4.8	
**Male adults (≥18 y, *N* = 14)**			
Age (y)		38.5 ± 19.8	
BMI (kg/m^2^)	<20^1^	19.0 ± 0.6	100% (14)
FFMI (kg/m^2^)	<17^1^^,^^2^	16.1 ± 1.1	71% (10)
ALM (kg)	<21.4^1^ or <20^3^	22.1 ± 3.7	43% (6)
ALMI (kg/m^2^)	<7.26^1^ or ≤7.0^3^	7.2 ± 0.6	36% (5)
ALMI adjusted for BMI (ALM/BMI)	<0.725^1^	1.2 ± 0.17	0% (0)
Grip strength (kg)	<27^3^	27.6 ± 7.7	36% (5)
PBF (%)	N/A	15.4 ± 4.1	
**Children & Adolescents (<18 y,***N* = **25)**			
Age (y)		14.0 ± 3.0	
BMI (kg/m^2^)		15.9 ± 1.4	
BMI z-score	Mild: −1 to −1.9^4^^,^^5^ Moderate: −2 to −2.9 Severe: <−3	−1.77 ±.73	72% (18) 16% (4) 8% (2)
%mBMI	Mild: 80–90^5^ Moderate: 70–80 Severe: < 70	84.2 ± 4.1	72% (18) 32% (8) 8%(2)
FMI z-score FFMI z-score	<−2 z-score 6	−1.98 ±.82 −1.02 ±.62	52% (13) 8% (2)

Abbreviations: %mBMI, percent of median BMI for age and sex; ALM, Appendicular Lean Mass; ALMI, Appendicular Lean Mass Index defined as ALM/height2; ALMI/BMI, Appendicular Lean Mass Index adjusted for BMI; DXA, Dual X-ray absorptiometry; FFMI, Fat-Free Mass Index; NA, not available; NHOPI, Native Hawaiian or Pacific Islander; PBF, Percent Body Fat.

1 ESPEN consensus diagnostic criteria for malnutrition [Cederholm et al. 2015] [9]

2 Global Leadership Initiative on Malnutrition consensus criteria [Cederholm 2019] [8]

3 Sarcopenia: Revised European consensus on definition and diagnosis [Cruz-Jentoft et al. 2019] [14]

4 Consensus of the Academy of Nutrition and Dietetics and American Society for Parenteral and Enteral Nutrition on pediatric malnutrition [Becker et al 2014] [38]

5 Medical management of restrictive eating disorders [Society for Adolescent Medicine 2022] [19]

6 Standardized body composition measurements to detect malnutrition risk in children with chronic conditions [Lara-Pompa et al. 2020] [13]

### Agreement between 3DO and DXA methods

**FIGURE 1:**
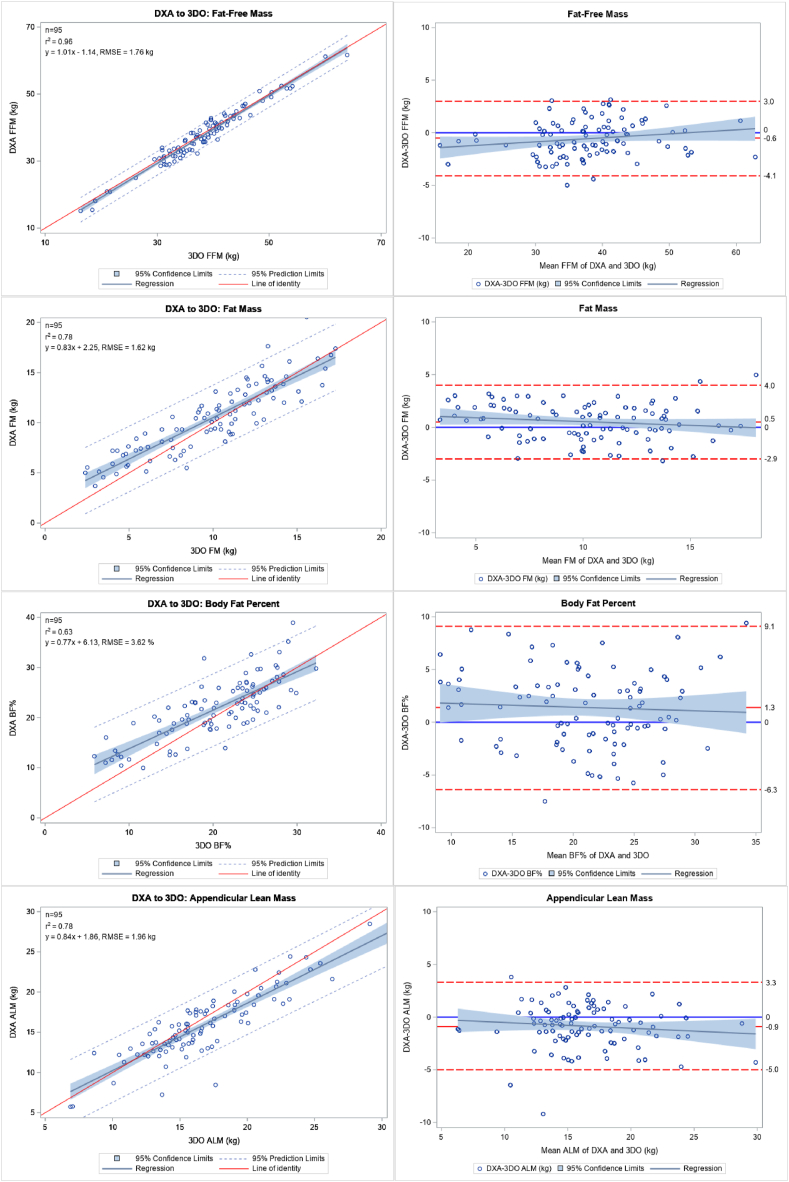
Linear regression plots with *R*^*2*^, regression equation and Root Mean Square Error (RMSE), and Bland-Altman plots for comparison of three-dimensional optical (3DO) imaging to dual-energy X-ray absorptiometry (DXA) in all study participants (*N* = 95). Abbreviations: fat-free mass (FFM), fat mass (FM), percent body fat (PBF), and appendicular lean mass (ALM).

Table 2 shows the difference and agreement between DXA and 3DO for the whole study population. Concordance between the 2 methods was 0.97 for FFM, 0.87 for FM, 0.77 for PBF, and 0.86 for ALM. These relationships are illustrated as linear regressions with corresponding Bland-Altman plots in Figure 1. Table 3 shows the number of participants identified as meeting the criteria for malnutrition and sarcopenia (as defined in Table 1) by DXA and 3DO. DXA, the criterion method, identified 80% of adult participants as having low FFMI consistent with malnutrition and 44% with low ALM consistent with sarcopenia. In the children, over half had FMI z-scores below −2 z-score threshold used to identify malnutrition in children and adolescents with chronic illnesses [13], including eating disorders [43]. For all malnutrition and sarcopenia criteria met by DXA, 3DO identified 95% of cases (165/173).

**TABLE 2:** Assessment of mean difference and agreement of 3DO and DXA (criterion) by variable for full study sample (N *=* 95)

Variable	Measurement *(mean ± SD)*		Difference *(mean ± SD)*	Correlation	
	*DXA*	*3DO*		R^2^	Lin’s CCC (95% CI)
FFM (kg)	37.7 ± 8.3	38.2 ± 8.0^1^	−0.6 ± 1.8	0.96	0.97 (0.96, 0.98)
FM (kg)	10.4 ± 3.5	9.9 ± 3.7^1^	0.5 ± 1.7	0.78	0.87 (0.82, 0.91)
PBF (%)	21.8 ± 5.9	20.4 ± 6.1^1^	1.4 ± 3.9	0.63	0.77 (0.68, 0.84)
ALM (kg)	16.0 ± 4.1	16.8 ± 4.4^1^	−0.9 ± 2.1	0.78	0.86 (0.80, 0.91)

Abbreviations: 3DO, 3-dimensional optical imaging; ALM, Appendicular Lean Mass; CCC, Correlation Coefficient indicates concordance between methods; DXA, Dual X-ray absorptiometry; FFM, Fat-Free Mass; PBF, Percent Body Fat.

1 3DO mean significantly differs from DXA mean by paired t-test (*P* < 0.05).

**TABLE 3:** Proportion of adults meeting phenotypic criteria used in the diagnosis of malnutrition and sarcopenia

	% (No.) meeting criteria^1^ by DXA	% (No.) meeting criteria∗ by 3DO	Expected agreement %	Observed agreement %	Κ^5^
All adults (age 18 and older, *N* = 70)					
Malnutrition criteria					
Low FFMI (kg/m^2^)^2^^,^^3^	80 (56)	84 (59)	52.2	90.5	0.80
Low ALM (kg)^2^	37 (26)	27 (19)	63.6	88.4	0.68
Low ALMI (kg/m^2^)^2^	26 (18)	24 (17)	69.9	86.3	0.54
Low (ALM/BMI)^2^	1.4 (1)	4 (3)	95.9	97.8	0.49
Sarcopenia criteria^4^					
Low ALM (kg)	44 (31)	54 (28)	57.1	90.5	0.78
Low ALMI (kg/m^2^)	37 (26)	33 (23)	61.7	84.2	0.58
Children and adolescents (age <18 y, *N* = 25)					
Low FMI z-score	52 (13)	56 (14)	75.6	92.6	0.70
Low FFMI z-score	8 (2)	8 (2)	84.8	88.4	0.24

Abbreviations: 3DO, 3-dimensional optical imaging; ALM, Appendicular Lean Mass; ALMI, Appendicular Lean Mass Index defined as ALM/height^2^; ALMIBMI, Appendicular Lean Mass Index adjusted for BMI; DXA, Dual X-ray absorptiometry; FFMI, Fat-Free Mass Index

1 Criteria for malnutrition and sarcopenia defined in Table 1 according to:

2 ESPEN consensus diagnostic criteria for malnutrition [Cederholm et al. 2015] [9]

3 Global Leadership Initiative on Malnutrition consensus criteria [Cederholm 2019][8]

4 Sarcopenia: revised European consensus on definition and diagnosis [Cruz-Jentoft et al. 2019][14]

5 (**Κ)** Cohen’s Kappa statistical test provides the expected and observed agreement and interrater reliability: <0 No agreement; 0 —.20 Slight;.21 —.40 Fair;.41 —.60 Moderate;.61 —.80 Substantial;.81–1.0 Perfect [Landis and Koch, 1977] [41]

Interrater reliability between DXA and 3DO is shown in Table 3. The agreement was approaching perfect for identifying malnutrition by low FFMI (Κ = 0.80) and sarcopenia by low ALM (Κ = 0.78). Agreement was substantial for identifying malnutrition by low ALM (Κ = 0.68) and moderate for low ALMI (Κ = 0.54). In children, agreement for identifying low FMI z-score was substantial (Κ = 0.70). In multivariate linear regressions, lower FFMI and lower ALMI by 3DO were independent predictors of lower grip strength (Β [confidence interval [CI]: −2.3 [−.9, −3.7], *P* = 0.002 and Β [CI]: −4.3 [-2.5, −6.0], *P* < 0.001) when adjusted for BMI and age.

### Eating disorder participant subset

Table 4 shows the characteristics of the 15 adolescents and young adults who were referred through the Eating Disorders Program. These participants comprised 16% (15/95) of the study population and included 8 young adults aged 18–25 y and 7 children and adolescents, as shown in Table 1. Average weight loss was ∼16% body mass over 1 y, which is considered severe [19,38]. Based on BMI criteria [19], these patients were mildly malnourished on average (80%–90% mBMI); 4 met the criterion for moderate malnutrition (70%–80% mBMI) and 1 met severe malnutrition (<70% mBMI). To examine whether participants enrolled in the Eating Disorder Program were representative of the larger sample of participants with low BMI included in this study, we compared key characteristics (Supplementary Table 1). Comparing the *N* = 8 young adults (18 – 25 y old) from the eating disorder subset to the *N* = 11 young adults in the larger study sample, age and BMI did not differ**.** However, FFMI was significantly lower (13.5 ± 1.3 compared with 15.3 ± 1.9, *P* = 0.04), with a tendency toward lower ALM (15.3 ± 2.5 compared with 19.5 ± 5.6, *P* = 0.06), lower ALMI (5.6 ±.75 compared with 6.6 ± 1.1, *P* = 0.06), lower grip strength (14.8 ± 6.4 compared with 24.4 ± 8.8, *P* = 0.07), and *higher* PBF (24.0 ± 3.5 compared with 18.2 ± 7.3, *P* = 0.06). The *N* = 7 children and adolescents in the eating disorder subset were significantly older than the *N* = 18 in the larger study sample (15.9 ± 0.5 compared with 13.3 ± 3.3 y, *P* = 0.04); however, %mBMI, handgrip, and z-scores of BMI, FFMI, and FM did not differ (all *P* > 0.10).

**TABLE 4:** Weight history in Eating Disorders Program participants, *N* = 15

		Mean ± SD	% (No.) meeting criterion^1^
Adults (≥18 y, *N* = 8)			
Age (y)		20.1 ± 2.8	
BMI (kg/m^2^)	<20 1	17.8 ± 2.1	75 (6)
%mBMI	Mild: 80–90 ^5^ Moderate: 70–80 Severe: < 70	82.2 ± 1.0	25 (2) 38 (3) 13 (1)
FFMI (kg/m^2^)	<15 (female); 20 (male) 1^,2^	13.4 ± 1.0	88 (7)
ALM (kg)	<14.1 (female); 21.4 (male) 1	15.3 ± 2.5	50 (4)
	<15 (female); 20 (male) ^3^		63 (5)
ALMI (kg/m^2^)	<5.25 (female); 7.26 (male) 1	5.7 ± 0.8	38 (3)
	<5.5 (female); 7.0 (male) ^3^		50 (4)
ALM/BMI	<0.591 1	0.85 ± 0.05	0
Grip strength (kg)	<16 (female) <27 (male) ^3^	14.8 ± 6.4	25 (2)
PBF (%)	N/A	23.9 ± 3.5	
Children & adolescents <18-y old (*N* = 7)			
Age (y)		15.9 ±.5	
BMI (kg/m^2^)		17.2 ±.9	
BMI z-score	Mild: −1 to −1.9 ^4,5^ Moderate: −2 to −2.9 Severe: <−3	−1.44 ±.5	57 (4) 14 (1) 0
%mBMI	Mild: 80–90 ^5^ Moderate: 70–80 Severe: <70	84.2 ± 4.1	86 (6) 14 (1) 0
PBF z-score	<−2 z-score ^6^	−1.10 ± 1.1	29 (2)
FFMI z-score	<−2 z-score ^6^	−1.22 ±.6	13 (1)
Historical weight (prior to study visit)			
Weight loss			
Highest BMI (kg/m^2^)		19.4 ± 2.4	
Highest %mBMI (%)		94.1 ± 10.3	
Lowest BMI (kg/m^2^)		16.1 ± 2.0	
Lowest %mBMI (%)		77.0 ± 8.3	
Total weight loss (highest to lowest, kg)		8.3 ± 6.5	
Percent of body weight lost		15.7 ± 12.1	
Duration of weight loss (mo)		11.7 ± 11.0	
Rate of loss (kg/mo)		0.29 ±.41	
Weight regain			
Weight regained by study visit (kg)		4.5 ± 3.5	
Weight regained as a percent of initial loss (%)		46.1 ± 78.3	
Duration of weight gain (mo)		6.5 ± 8.2	
Rate of weight gain (kg/mo)		1.5 ± 1.9	

Abbreviations: %mBMI, percent of median BMI for age and sex; ALM, Appendicular Lean Mass; ALMI, Appendicular Lean Mass Index defined as ALM/height^2^; ALM/BMI, Appendicular Lean Mass Index adjusted for BMI; FFMI, Fat-Free Mass Index; PBF, Percent Body Fat.

1 Criteria for malnutrition and sarcopenia are defined in Table 1 with references in footnotes 1-6

### Associations between weight history and body composition:

**FIGURE 2:**
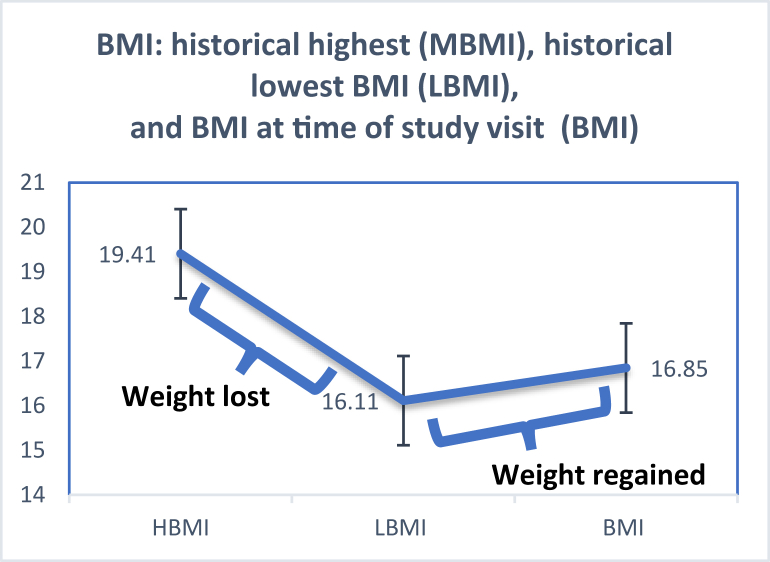
Weight History among Eating Disorder Program participants (*N* = 15) with mean ± SD historical highest BMI prior to illness (HBMI); lowest BMI around the time of referral (LBMI); and measured BMI at the study visit (BMI).

Participants referred through the Eating Disorder Program underwent body composition testing during various phases of weight recovery. Figure 2 illustrates the trajectory from the historically highest BMI prior to illness to the lowest BMI recorded and BMI measured at the study visit. On average, these patients lost ∼8 kg prior to referral and regained about half of that by the study visit (see Table 4 for BMI and weight gain/loss data). Table 5 shows relationships between weight changes (percent of body mass lost or percent regained) and body composition, both unadjusted and adjusted for highest weight (highest historical %mBMI). Independent of the highest weight, a greater percentage of body weight lost prior to the study visit predicted lower FFM, FM, and ALM by both DXA and 3DO; a greater percentage of weight regained prior to the study predicted higher PBF by both methods.

**Table 5:** Associations between weight history body composition in Eating Disorder Program participants, *N =* 15

Outcome	Weight lost (%)		Weight regained (%)	
	Unadjusted Β (CI)	Adjusted^3^ Β (CI)	Unadjusted Β (CI)	Adjusted^3^ Β (CI)
DXA				
FFM (kg)	−0.27 (−0.45, −0.08)^2^	−0.38 (−0.51, −0.25)^2^	−0.03 (−0.06, 0)	−0.03 (−0.06, 0)
FM (kg)	−0.09 (−0.21, 0.04)	−0.13 (−0.26, −0.01)^1^	0.01 (−.01, 0.03)	0.01 (0.01, 0.03)
PBF (%)	−0.03 (−.022, 0.15)	−0.48 (−0.26, 0.17)	0.03 (0, 0.05)^1^	0.03 (0, 0.05)^1^
ALM (kg)	−0.13 (−0.23, −0.03)^1^	−0.19 (−0.26, −0.12)^2^	−0.02 (−0.03, 0)^1^	−0.02 (−.03, 0)^1^
3DO				
FFM (kg)	−0.31 (−0.47, −0.13)^2^	−0.40 (−0.53, −0.27)^2^	−0.03 (−0.07, 0)^1^	−0.03 (−0.06, 0)
FM (kg)	−0.06 (−0.18, 0.07)	−0.11 (−0.23, 0)	0.01 (−0.01, 0.03)	0.01 (−0.01, 0.04)
PBF (%)	−0.02 (−0.16, 0.21)	−0.01 (−0.21, 0.20)	0.03 (0.01, 0.05)^2^	0.03 (0.01, 0.05)^2^
ALM (kg)	−0.19 (−0.30, −0.08)^1^	−0.24(−0.35, −0.12)^2^	−0.02 (−0.04, 0)	−0.02(−0.04, 0)

1 Independent variable (percent weight lost or gained) is significantly associated with outcome as listed at *P* < 0.05

2 Indicates *P* < 0.01

3 Beta coefficient (**Β)** and Confidence Interval (CI) from a multivariable model; adjusted model includes highest historical weight (as a percent of median body mass index)

## Discussion

Our findings support the utility of 3DO imaging as a tool to assess body composition in patients with low BMI. Concordance between 3DO and DXA was excellent for the estimation of lean mass and strong for fat mass and ALM. These findings are consistent with our prior studies establishing the accuracy and precision of 3DO in approximately 1400 adults [34,31,35], children, and adolescents [30] of diverse age, ethnicity, and BMI enrolled in the parent Shape Up! clinical trials. However, building on prior studies, here, we showed that 3DO can accurately estimate body composition in participants with low BMI who met other phenotypic criteria for malnutrition [8] and sarcopenia [14]. Further, 3DO showed excellent agreement with DXA for identifying participants meeting these criteria. These findings have broad relevance for adults [1,44] and children [38] with malnutrition, which increases complications and predicts poor treatment outcomes across a range of illnesses. In this study, we demonstrate specific relevance for patients with malnutrition secondary to restrictive eating disorders, who have medical complications and impaired physical functioning related to losses in all tissue compartments.

New recommendations have added body composition, along with BMI and weight loss, as a key phenotypic indicator of malnutrition in adults [8,9,11,12,14], and the field of pediatrics is moving in the same direction [[11], [12], [13],^43^,^45^,^46^]. Studies implementing the Global Leadership Initiative on Malnutrition (GLIM) criteria demonstrate that the inclusion of body composition improves the prediction of functional outcomes and mortality in chronic illness (including cardiovascular disease [2], chronic obstructive pulmonary disease [3], and cancers [4]), as well as hospitalized adults [47,48] and children [13]. Lean mass assessment appears to be the most salient addition to the phenotypic indicators [3,4,13]. Low FFM reflects peripheral wasting as well as loss of visceral protein in patients with malnutrition, including reductions in myocardial, hepatic, renal, and splenic mass [49], whereas low ALM is favored as a more specific indicator of low skeletal muscle mass related to the impaired function and poor outcomes of sarcopenia.[14] The high concordance between 3DO and DXA for lean mass and near-perfect agreement for the identification of malnutrition by low FFMI and sarcopenia by low ALM underscores the potential value of 3DO for assessment.

As an additional measure of agreement with DXA, we examined whether 3DO could identify criteria for malnutrition and sarcopenia in participants enrolled for low weight but not a particular diagnosis. “Constitutional thinness” has been well characterized as a benign state of low lean mass [50]. Although such individuals are not of clinical concern, they cannot be discerned by BMI alone. This is illustrated by our finding that virtually all participants had low BMI, and 80% had low lean mass, yet less than half met the more the rigorous sarcopenia thresholds for low ALM. Among 31 cases of sarcopenia by low ALM identified by DXA, 3DO identified 91% with near-perfect agreement between methods. Further, we showed that low FFMI and ALMI by 3DO were associated with low handgrip strength independent of BMI. Handgrip is included in the assessment of sarcopenia because it is a strong predictor of patient outcomes that is correlated with other measures of strength and can be measured in bed [10]. Our findings indicate that 3DO is sensitive enough to discern between nonpathologic low lean mass [51] and reduced lean mass associated with impaired physical function.

3DO is poised to fill the pressing need for an accessible and reliable clinical tool to assess malnutrition and monitor the progress of nutritional rehabilitation. Since the 2019 GLIM publication, 79 studies have implemented the new recommendations, but only about half employed all criteria, including body composition [52]. Lack of access to DXA has been cited as a barrier [11] because instrumentation is large and subject to radiation safety standards. Further, concern for cumulative exposure limits serial measures to 4 per year in adults [29] and 2 per year in pediatric patients [28] unless medically necessary. On the other hand, 3DO emits no ionizing radiation and can be used for repeated assessment to monitor the progress of interventions. We recently reported that 3DO was as sensitive as DXA in detecting changes in FFM, FM, and ALM in response to interventions as short as 3–23 wks [35]. In the context of the present cross-sectional design, we utilized weight history in a subset of patients referred to our Eating Disorder Program to investigate the potential for 3DO to capture changes in body composition because of recent weight changes. When accounting for weight prior to illness, a greater percentage of body mass lost predicted lower LM, FM, and ALM at the time of the study visit by both 3DO and DXA. A greater percentage of weight *regained* by the time of the study visit predicted significantly greater PBF using both methods, regardless of historical weight. These findings comport with DXA and MRI studies of body composition in malnourished patients with AN, showing that weight is lost from all compartments during starvation (particularly the extremities) and regained disproportionately as adipose [20,[53], [54], [55]]. Together with our recent study of obese participants undergoing prospective interventions [35], these findings support the use of 3DO to detect short-term changes in body composition during the course of nutritional rehabilitation.

Body composition standards are urgently needed to assess malnutrition in patients with eating disorders. In the past, low weight at presentation was used to indicate the extent of malnutrition [37,[56], [57], [58]] and monitor the progress of nutritional rehabilitation [[59], [60], [61]]. However, in 2013, weight was removed as a diagnostic criterion for AN, and a new diagnosis, atypical AN, was developed to describe AN following “significant” weight loss but not low BMI. Patients with atypical AN now comprise ∼1/3 of eating disorder program inpatient censuses [[62], [63], [64]]. We previously reported that such patients had medical complications, laboratory abnormalities, and amenorrhea that were just as severe as patients with AN and low BMI [65]. These developments reflect a rapidly diversifying patient population [66] and expose the limitations of overreliance on weight to gauge clinical concern. Our findings support the potential for 3DO to fill this void.

Whereas agreement with DXA was strong for lean, fat, and ALM compartments, a key limitation was that agreement for PBF was lower than previously reported [34,30,31,35]. This is likely due to the range compression in this population with low BMI and average FM of ∼10 Kg. For context, among the 407 participants in the parent Shape Up! trial, men had a mean (SD) BMI of 28.4 (6.2) kg/m^2^ and 20.9 (10.7) kg of fat mass; women had a BMI of 27.4 (5.5) kg/m^2^ and 19.4 (9.5) kg of fat. In that population with a larger overall mass and range of fat tissue, an agreement between 3DO and DXA was much better (*R*^*2*^ = 0.93 and 0.94 in men and women, respectively) [31]. Additionally, although the present study showed strong overall agreement between 3DO and DXA, a marked increase in error in these participants with low BMI who had low PBF (< 20%), evident in Figure 1, contributed heavily to poorer overall concordance. This problem of variance can be addressed in the future by enrolling more participants in the lower BMI range. Although fat mass is not included in the new definitions of malnutrition, it has important clinical implications for patients with AN, and therefore, the accuracy of FM estimation by 3DO warrants attention for future refinements. Regardless, the high precision of 3DO allows it to monitor small and large FM *changes* over time. [35] Persistently low fat mass during weight recovery from AN, such as that seen in female athletes, increases the risk of relapse [26,27] and persistent amenorrhea. A minimal threshold of PBF to support menstruation is estimated at around 21% [67]. Although the female adults in the present study were above this threshold, this cannot be interpreted to mean that nutritional status was sufficient. Indeed, patients with atypical AN are just as likely to have hypogonadism evidenced by amenorrhea [65] despite their greater fat mass [68]. Another limitation of the current study was the small size of the eating disorder subset. This subset was valuable for examining the clinical utility of 3DO in a patient population with documented weight loss. However, weight loss was assessed retrospectively by a weight history questionnaire that did not assess the etiologic contributors (e.g., reduced dietary intake) emphasized in new malnutrition recommendations [8,58]. Longitudinal studies are needed to investigate body composition by 3DO during nutritional rehabilitation in patients with malnutrition secondary to eating disorders.

## Appendix A Supplementary data

Multimedia component1 Multimedia component1
