# Supplementary material for: Cross-sectional assessment of body composition and detection of malnutrition risk in participants with low body mass index and eating disorders using 3D optical surface scans
Source: Am J Clin Nutr. 2023 Aug 19;118(4):812–21. doi: 10.1016/j.ajcnut.2023.08.004 (PMC10797509; doi:10.1016/j.ajcnut.2023.08.004)
Supplement: Multimedia component1 [file mmc1.docx]

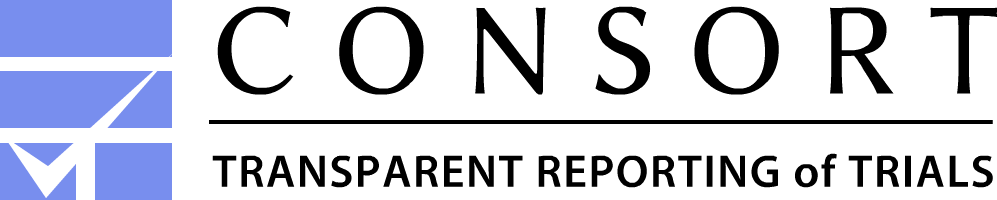


**CONSORT 2010 Flow Diagram**

## Enrollment

Shape Up! Adults Total study enrollment (n=883)

- UCSF (n=230)
- PBRC (n=345)
- UHCC (n=127)
- Ancillary (n=181)

Shape Up! Kids Total study enrollment (n=465)

- UCSF (n=22)
- PBRC (n=269)
- UHCC (n=174)

Exclusion (n=1253)

- BMI > 20 kg/m^2^ (n=1017)
- Ancillary participants for separate study protocol (n=181)
- Dropouts (n=7)
- Missing or unusable Fit3D scan (n=48)
- Invalid DXA scan (n=13)

Analyzed (Total n=95)

- n = 70 adults
- n = 25 children

## Analysis

**Supplemental Figure 1**: CONSORT flow diagram for enrollment in the Shape Up! Adults and Kids studies (NIH R01DK109008 and R01DK111698) as of this publication.

Abbreviations: Body Mass Index (BMI); University of California, San Francisco (UCSF); University of Hawaii Office of Research Compliance (UHCC); and Pennington Biomedical Research Center (PBRC)

**Supplemental Table 1:** Comparison of participants enrolled through Eating Disorders Program to the larger sample of participants with low BMI.

| **Characteristics at study visit** | **Participants enrolled through Eating Disorder Program** | **Low BMI participants** | **p-value^** |
| --- | --- | --- | --- |
| **Young adults (age 18-25 years)** | **N=8** | **N=11** |  |
| Age (years) | 20.1 ± 2.8 | 21.5 ± 2.3 | 0.27 |
| BMI (kg/m^2^) | 17.9 ± 2.1 | 18.7 ± 1.0 | 0.28 |
| FFMI (kg/m^2^) | **13.5 ± 1.3** | **15.3 ± 1.9** | **0.04** |
| ALM (kg) | **15.3 ± 2.5** | **19.5 ± 5.6** | **0.06** |
| ALMI (kg/m^2^) | **5.6 ± 0.75** | **6.6 ± 1.1** | **0.06** |
| Grip strength (kg) | **14.8 ± 6.4** | **24.4 ± 8.8** | **0.07** |
| PBF (%) | **24.0 ± 3.5** | **18.2 ± 7.3** | **0.06** |
| **Children & Adolescents (age <18 years)** | **N=7** | **N=18** |  |
| Age (years) | **15.9 ± 0.5** | **13.3 ± 3.3** | **0.04** |
| BMI z-score | -1.5 ± 0.4 | -1.9 ± 0.8 | 0.27 |
| %mBMI | 84.2 ± 5.2 | 82.3 ± 5.6 | 0.43 |
| FMI z-score | -1.7 ± 0.7 | -2.1 ± 0.9 | 0.34 |
| FFMI z-score | -0.85 ± 0.6 | -1.1 ± 0.6 | 0.44 |
| Grip strength (kg) | 16.3 ± 6.4 | 16.4 ± 9.9 | 0.99 |

Values are shown are mean±SD

^ P-value for unpaired t-test of group means

Abbreviations: Body Mass Index (BMI); percent of median BMI for age and sex (%mBMI); Fat Mass Index (FMI); Fat Free Mass Index (FFMI); Appendicular Lean Mass (ALM); Appendicular Lean Mass Index (ALMI) defined as ALM/height^2^; Percent Body Fat (PBF).
